# SNP Analysis and Whole Exome Sequencing: Their Application in the Analysis of a Consanguineous Pedigree Segregating Ataxia

**DOI:** 10.3390/microarrays4040490

**Published:** 2015-10-23

**Authors:** Sarah L. Nickerson, Renate Marquis-Nicholson, Karen Claxton, Fern Ashton, Ivone U. S. Leong, Debra O. Prosser, Jennifer M. Love, Alice M. George, Graham Taylor, Callum Wilson, R. J. McKinlay Gardner, Donald R. Love

**Affiliations:** 1 Diagnostic Genetics, LabPLUS, Auckland City Hospital, P.O. Box 110031, Auckland 1148, New Zealand; E-Mails: Snickers@adhb.govt.nz (S.L.N.); KClaxton@adhb.govt.nz (K.C.); FernA@adhb.govt.nz (F.A.); IvoneL@adhb.govt.nz (I.U.S.L.); DProsser@adhb.govt.nz (D.O.P.); JLove@adhb.govt.nz (J.M.L.); AliceG@adhb.govt.nz (A.M.G.); 2 Centre for Translational Pathology, University of Melbourne, Corner Grattan Street and Royal Parade, Parkville, Victoria 3010, Australia; E-Mails: Renate.mn@gmail.com (R.M-N.); graham.taylor@unimelb.edu.au (G.T.); 3 Paediatric Metabolic Service, Starship Children’s Hospital, Auckland 1148, New Zealand; E-Mail: callumw@adhb.govt.nz; 4 Clinical Genetics Group, Dunedin School of Medicine, University of Otago, Dunedin 9016, New Zealand; E-mail: macgardner@gmail.com

**Keywords:** *SACS*, ARSACS, sacsin, autosomal recessive ataxia, microarray, exome sequencing, homozygosity, consanguinity

## Abstract

Autosomal recessive cerebellar ataxia encompasses a large and heterogeneous group of neurodegenerative disorders. We employed single nucleotide polymorphism (SNP) analysis and whole exome sequencing to investigate a consanguineous Maori pedigree segregating ataxia. We identified a novel mutation in exon 10 of the *SACS* gene: c.7962T>G p.(Tyr2654*), establishing the diagnosis of autosomal recessive spastic ataxia of Charlevoix-Saguenay (ARSACS). Our findings expand both the genetic and phenotypic spectrum of this rare disorder, and highlight the value of high-density SNP analysis and whole exome sequencing as powerful and cost-effective tools in the diagnosis of genetically heterogeneous disorders such as the hereditary ataxias.

## 1. Introduction

Autosomal recessive cerebellar ataxias (ARCAs) are a complex group of disabling neurodegenerative disorders that manifest predominantly in childhood and early adulthood [1]. Despite increasing knowledge regarding the molecular basis of ARCA, a considerable number of patients remain without a specific diagnosis [2]. The diagnosis is challenging due to both genotypic and phenotypic heterogeneity [1]. More than 30 genes/loci have been associated with over 20 different clinical forms of ARCA [3]. Phenotypic variability in the expression of cerebellar impairment, including atypical phenotypes and overlapping clinical features, further complicates the picture [2]. The delineation of the precise clinical features ascribed to each ARCA remains under debate, hence complicating, yet also necessitating, a conclusive molecular diagnosis.

Mutations causative of different forms of ARCA are frequently located in genes of particularly large coding capacity (e.g., *SYNE1* (NM_182961) 26 kb in 145 coding exons [4], *SACS* (NM_014363) 14 kb in 9 exons [5] and *ATM* (NM_000051) 9 kb in 62 coding exons [6]). Sanger-based (dideoxy) DNA sequencing is the gold standard for detecting mutations at the base pair level, but it is costly on an individual gene basis, and given the number and size of associated genes, its use in the investigation of unidentified ARCA is limited.

Here, we describe the application of high-density single nucleotide polymorphism (SNP) analysis and whole exome sequencing (WES) in the investigation of two Maori siblings presenting with apparent ARCA.

## 2. Case Presentation

The two affected siblings (V:11 and V:12, Figure 1), now deceased, were of Maori and English ancestry, and there were twelve unaffected sibs. Their parents were first cousins. The siblings share a similar progressive clinical history, characterised primarily by cerebellar ataxia, peripheral neuropathy, and pyramidal tract signs. The initially suspected diagnosis was Friedreich ataxia, but *FXN* gene testing returned normal results.

The index case (V:11), now deceased, experienced onset of lower limb weakness in his early twenties. By his late forties he was only able to walk very short distances with a frame, reliant predominantly on a wheelchair. At this stage, he was also affected by upper limb ataxia, being unable to perform fine motor movements such as fastening buttons.

Clinical examination at the age of fifty-two years revealed dysarthria, nystagmus on lateral gaze, and an intention tremor. He had marked wasting of the intrinsic muscles of the hands and distal quadriceps. His tone was normal throughout. Upper limb power was normal, except the small muscles of the hands; however, he was now wheelchair bound with truncal weakness and reduced power of the lower limbs (4/5 at the hips and knees, and minimal movement at the ankles). Tendon reflexes were absent in the lower limbs, except for a flicker at the knees. Plantars were extensor. Sensation was intact, but vibration sense was absent in the lower limbs. By sixty-one years of age, his clinical features had further progressed. Power had also slightly reduced in the upper limbs, with absent reflexes other than a small flicker of the triceps. Tone remained normal throughout. Fundoscopy revealed no abnormalities. He died from pneumonia at the age of sixty-five years.

**Figure 1:**
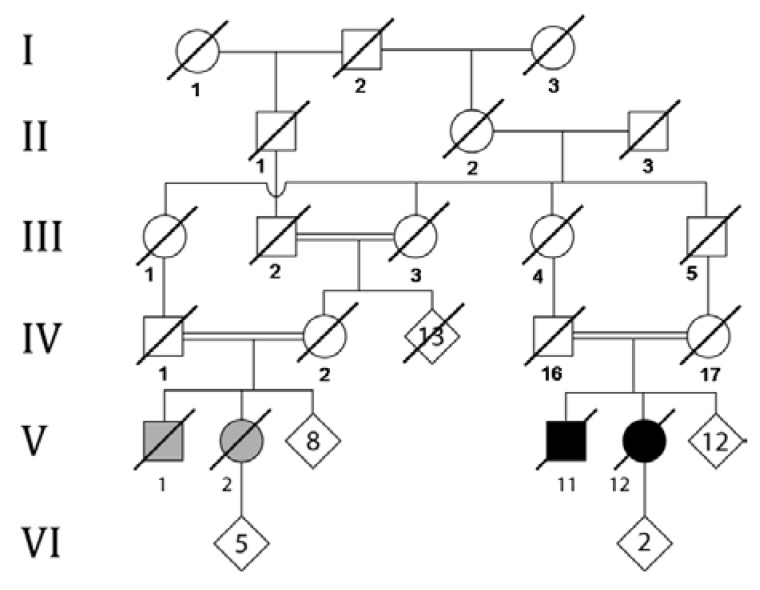
Abbreviated pedigree of the family. Of the founders in generation I, I:2 was English, and I:1 and I:3 both Maori; “marrying-in” spouses in generations II and III were also Maori. Cousin marriages are shown as double lines. The siblings diagnosed with autosomal recessive spastic ataxia of Charlevoix and Saguenay (ARSACS) are V:11 and V:12 (filled symbols). The siblings V:1 and V:2 (grey symbols) are suspected, but not proven, to have had the same condition. Crossed symbols indicate deceased individuals.

His sister (V:12), now also deceased, was at the time of our study a sixty-four year old woman, and shared a similar clinical history. Examination in her mid-late thirties had revealed moderate dysarthria, dysdiadochokinesia, and a slight intention tremor. Her gait was ataxic with a positive Romberg sign. She was able to walk unaided; lower limb tone was normal, but there was moderate symmetrical weakness, absent ankle reflexes, and extensor plantars. Upper limb tone, power and sensation were normal, but tendon reflexes were reduced.

By her late fifties, examination revealed a progression in her clinical features. She had become wheelchair-bound, with clear wasting from the mid thighs distally. Lower limb tone was normal, all reflexes were absent, and plantars were extensor. Vibration sense was grossly intact at the lower thigh but absent distally. She had severe Achilles contracture. In the upper limbs there was an impression of loss of bulk in the forearms, and clear wasting of the thenar, first interosseous and intrinsic muscles. All reflexes were absent. Vibration sense remained intact in the upper limbs. Fundal examination was unremarkable, but there was occasional coarse nystagmoid movement on lateral gaze.

At the age of thirty-six years, A CT scan demonstrated widening of cortical sulci over both cerebral hemispheres, compatible with minor cerebral atrophy, some cerebellar atrophy with enlargement of the fourth ventricle and cisterna magna, and widening of subarachnoid spaces over the superior aspect of the cerebellum. Electromyography showed very low amplitude dispersed compound muscle action potentials in the lower limbs, slowing of all motor conduction velocities, and absent sensory nerve action potentials. Neurogenic motor unit potential changes and fibrillation in distal muscles were evident.

Two cousins of the probands (V:1 and V:2, Figure 1), also born of a consanguineous union, were suspected to have been affected, but were deceased prior to the availability of molecular analysis. Clinical records were available for one of these individuals, and described a remarkably similar phenotype to the probands, with the exception of an early childhood/congenital onset, including early cognitive impairment.

## 3. Molecular Studies

Genomic DNA was isolated from peripheral blood of the two affected siblings, together with an unaffected brother, using the Gentra Puregene blood kit according to the manufacturer’s instructions (Qiagen, Gaithersburg, MD, USA).

### 3.1. SNP Analysis

An Affymetrix Cytogenetics Whole-Genome 2.7 M Array was used for SNP analysis in the two affected siblings. This array consists of 2.7 million probes spaced across the genome, enabling high-resolution genome-wide interrogation and allelic discrimination. Genomic DNA (0.1 μg) was labelled using the Affymetrix Cytogenetics Reagent Kit (Affymetrix, Santa Clara, CA, USA), applied to the Affymetrix Cytogenetics 2.7 M array and scanned according to the manufacturer’s instructions (Affymetrix Cytogenetics Assay Protocol [7]). Data was analysed using the Affymetrix Chromosome Analysis Suite (ChAS) v1.0.1/na30.1 with the aid of the UCSC genome browser [8]. All genomic coordinates were taken from the human reference sequence hg18 (NCBI build 36.1). Our reporting policy is to omit copy number changes that do not contain genes, are well established polymorphisms, are losses smaller than 200 kb, or are gains smaller than 400 kb, unless associated with a gene of known clinical significance. Regions of long contiguous stretches of homozygosity (LCSH) greater than 5 Mb are detected. The array will not detect balanced alterations (translocations, inversions and insertions).

### 3.2. WES

WES was performed in the affected siblings, V:11 and V:12, and one unaffected sibling, in order to investigate the locus of the undelineated neurodegenerative hereditary ataxia. The Nextera Rapid Capture Exome kit (Illumina, San Diego, CA, USA) was used to target 214,405 coding exons (37 Mb), which comprise 98.3% of all RefSeq genes. Sequencing was performed using an Illumina HiSeq 2500, achieving a mean coverage of over100×. An in-house bioinformatics pipeline was used to process the data and generate an annotated variant call file. Further annotation and variant filtering were carried out using Variant Studio software (Illumina). Variants were annotated against all gene transcripts and reported against the HGNC recommended transcript.

### 3.3. Primer Design and Sanger-based Sequencing

Primers were designed to flank the relevant exon of the *SACS* gene, including 50 bp of the flanking intronic regions. The UCSC genome browser was used to obtain the reference transcript of the *SACS* gene (NM_014363.4) and its protein product (NP_055178.3). This website provides a direct link to ExonPrimer for the design of primers flanking coding exons. The primers were checked for underlying SNPs using the online software tool available from the National Genetic Reference Laboratory, Manchester [9]. After passing *in silico* tests, the primers were tailed with M13 sequences and synthesised by Integrated DNA Technologies (details available upon request).

Sanger-based sequencing was performed to confirm the research-based WES results and the traces were analysed using Variant Reporter™ Software v1.0 (Thermo Fisher Scientific, Cleveland, OH, USA) as described previously [10]. GenBank NM_014363.4 was used as the reference sequence, with cDNA number +1 corresponding to the A of the translation initiation codon (codon 1). Each sequence trace had a minimum trace score of 35, which corresponds to an average false base call frequency of 0.031%.

## 4. Results

**Figure 2:**
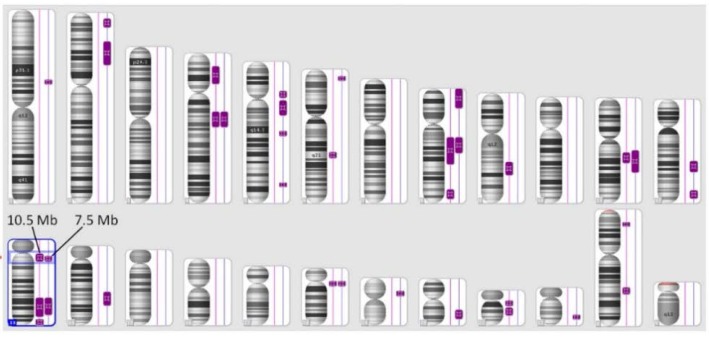
Regions of long contiguous stretches of homozygosity (LCSH) over 5 Mb in length identified by SNP analysis in individuals V:11 (right track) and V:12 (left track). Chromosomes 1 to 12 are depicted in order from top left to right; chromosomes 13 to 22, X and Y are depicted in order from bottom left to right. The arrowed region on chromosome 13 shows the location of the *SACS* gene, contained within the LCSHs of indicated sizes.

SNP analysis revealed nineteen regions of LCSH over 5 Mb in length in individual V:11, five of which were above 20 Mb long, and a total of fourteen regions of LCSH in individual V:12 over 5 Mb, including three above 20 Mb (Figure 2). Of the identified LCSH regions, six were common between the affected siblings (Table 1) and thus likely to harbour the locus for their recessively-inherited cerebellar ataxia. No clinically significant copy number changes were detected in either case.

**Table 1:** Regions of LCSH common to the affected siblings, as detected by SNP analysis.

Chromosome	Cytoband		Chromosome Coordinates (hg18)		Size (kb)
	Start	End	Start	End	
4	q13.3	q22.2	75,140,321	94,467,450	19,327
8	q12.2	q21.1	62,360,831	82,468,093	20,107
11	q13.3	q14.1	70,171,744	83,268,529	13,097
13	q12.11	q12.3	21,382,994	28,897,752	7,515
13	q22.3	q32.3	77,509,929	100,244,550	22,735
18	q11.1	q11.2	16,803,434	23,248,050	6,445
Total	-	-	-	-	89,226

A search of OMIM genes associated with ataxia and located within the regions of LCSH common to both affected siblings yielded seven candidate genes (Table 2); however, there was no single gene for which full phenotypic concordance could be seen.

**Table 2:** Candidate ataxia genes located in the regions of LCSH common to both affected siblings, identified by searching OMIM genes for the specific clinical feature “ataxia”. Abbreviations: AR, autosomal recessive; AR, autosomal dominant.

Gene	Phenotype	Inheritance
*COQ2*	Multiple-system atrophy	AR, AD
*GRID2*	Autosomal recessive spinocerebellar ataxia-18	AR
*TTPA*	Ataxia with isolated vitamin E deficiency	AR
*CYP7B1*	Autosomal recessive spastic paraplegia-5A	AR
*PEX2*	Peroxisome biogenesis disorder-5B	AR
*SACS*	Autosomal recessive spastic ataxia of Charlevoix-Saguenay	AR
*ATP8A2*	Cerebellar ataxia, mental retardation, and disequilibrium syndrome-4	AR

**Figure 3:**
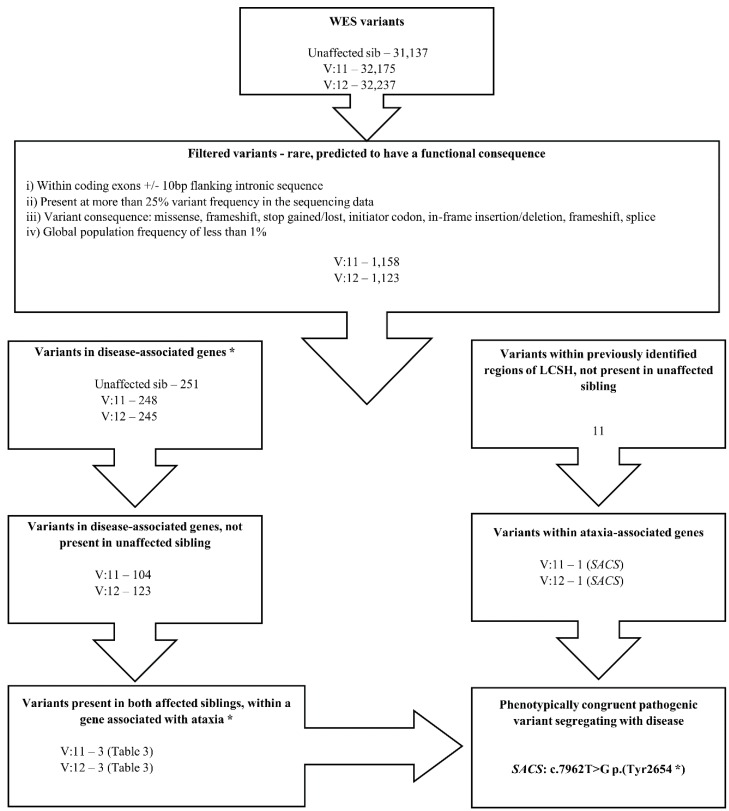
The pipeline used to select variants of potential clinical interest from exome sequencing. The right arm incorporates the homozygosity data obtained by SNP array; the left arm does not incorporate this information. * Based on Variant Studio annotation, OMIM, NCBI.

**Figure 4:**
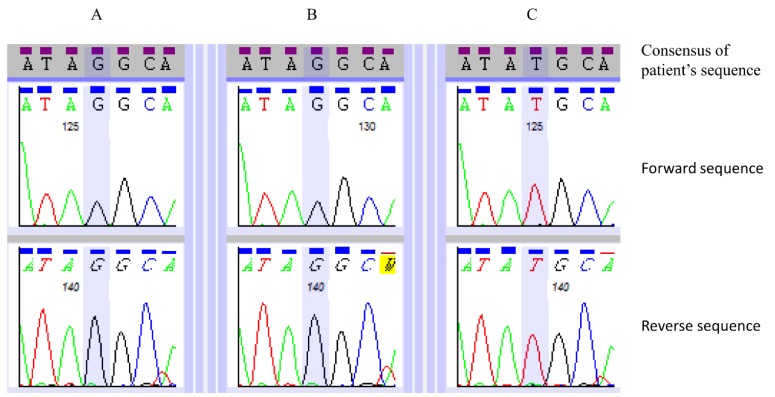
Sequence electropherograms showing the bidirectional traces of a region of exon 10 of the *SACS* gene. Apparent homozygosity for the variant c.7962T>G p.(Tyr2654 *) is seen in the two affected siblings (panels **A** and **B** representing V:11 and V:12, respectively). The variant was not detected in an unaffected sibling (panel **C**).

Rather than screen candidate ataxia genes found in the shared LCSH regions, we undertook a complementary WES study, with variant filtering as described (Figure 3), which yielded three possible variants (Table 3). The variant most concordant with the phenotype, most likely to be clinically significant, and located in one of the LCSH regions common to both affected siblings, was the *SACS* gene variant c.7962T>G p.(Tyr2654*). Sanger-based sequencing confirmed the apparent homozygosity of this variant in the two affected siblings (Figure 4).

**Table 3:** WES prioritised variants. Variant filtering was performed using an in-house bioinformatics pipeline and Variant Studio software (Illumina). * Data obtained from the ExAC database [11]. Abbreviations: Het, heterozygous; Hom, homozygous; MAF, minor allele frequency.

Gene	Nucleotide Change	Amino Acid Change	Variant State	Located in a Region of LCSH Common to Both Affected Siblings	MAF *	Comments
*CACNA1A*	NM_023035.2: c.793C>G	NP_075461.2: p.(Gln265Glu)	Het	No	0.0	Point mutations in *CACNA1A* are consistent with phenotype. Doesn’t fit apparent inheritance pattern in this pedigree.
*SACS*	NM_014363.4: c.7962T>G	NP_003045.2: p.(Tyr2654*)	Hom	Yes	0.0	Truncating mutation. Homozygous in both affected siblings. Consistent with phenotype.
*ZNF592*	NM_014630.2: c.3023G>A	NP_055445.2: p.(Arg1008Gln)	Het	No	4.523 × 10^−5^	Incomplete concordance with phenotype; AR disorder, single het variant detected only.

The nonsense variant, c.7962T>G p.(Tyr2654 *), has not, to our knowledge, been previously reported in the literature or mutation databases (Leiden Open Variation Database; LOVD; www.lovd.nl/3.0/home, Human Gene Mutation Database Professional; HGMD^®^ Pro) [12]; but, given that it results in premature termination of mRNA translation, it is predicted to be pathogenic. The Mutalyzer 2.0.13 website [13] predicts that this variant produces an amino acid sequence which is 58% of the length of that coded by the reference *SACS* gene transcript (Refseq accession number: NM_014363.4).

## 5. Discussion

ARSACS (OMIM 270550) is a complex neurodegenerative disorder caused by mutations in the *SACS* gene [5]. ARSACS was first described in individuals from the Saguenay-Lac-St-Jean area of north-eastern Quebec [14], where cases are predominantly due to two founder mutations: c.6594delT and c.5254C>T [15]. It is now well recognised that ARSACS is not limited to this region, but occurs worldwide [16,17,18,19,20,21,22,23]. The disorder is likely under-diagnosed and the true incidence remains unknown; after Freidreich ataxia and ataxia-telangiectasia, it may be one of the more frequently seen of the recessively-inherited ataxias [24].

ARSACS is characterised by progressive cerebellar ataxia, pyramidal tract signs, and a peripheral sensorimotor neuropathy predominantly affecting the lower limbs [14]. Progressive lower limb spasticity is considered a core clinical feature, and is associated with the preservation of tendon reflexes, except for ankle jerks [14,16]. The patients we describe here differ from the classical phenotype as their lower limb tone was normal and tendon reflexes were absent. Two other ARSACS families with a spasticity-lacking phenotype have been reported, but the genotypes in each case differed: c.987T>C and c.5988_9delCT [25,26]. As observed in our cases, these individuals displayed bilateral Babinski signs indicating pyramidal involvement. This highlights that spasticity is not a constant feature of ARSACS, and its absence should not exclude ARSACS as a differential diagnosis in cases of early-onset cerebellar ataxia.

Disease onset occurred in adulthood in the probands reported here, whereas in individuals originating from Quebec onset occurs between 12 and 18 months of age. Although cases of adult onset have been described outside of this region, childhood onset is the norm [27]. Early childhood/congenital onset was observed in a cousin of the probands (V:2, Figure 1); however, early cognitive impairment was also present. Her phenotype could be explained by a coincidental birth injury, or an effect of marked environmental deprivation, upon which the familial ARCA was superimposed; although we note also more recent reports of a proposed cognitive/psychological component *per se* of ARSACS [28,29], and it is speculative whether this may have been a factor.

Treatment for ARSACS remains largely symptomatic. Postmortem examination has demonstrated atrophy of the anterior cerebellar vermis associated with Purkinje cell death, small corticospinal tracts, and demyelination of both spinal cord corticospinal and posterior spinocerebellar tracts [30,31].

The *SACS* gene is located on chromosome 13q12.12 [32,33] and encodes an 11.7 kb protein, sacsin, which is highly expressed throughout the central nervous system, as well as in skeletal muscle and skin fibroblasts [5]. The carboxyl-terminus domain contains a “DnaJ” motif that binds the heat shock protein Hsc70 [34]. Its additional ubiquitin-like domain indicates that sacsin could play a role in linking the ubiquitin-proteosome pathway to the heat shock protein 70 machinery [34]. Recent studies in homozygous *Sacs* knockout (*Sacs*−/−) mice have revealed that an absence of sacsin leads to abnormal accumulation of non-phosphorylated neurofilament bundles in the somatodendritic regions of vulnerable neuronal populations and in ARSACS brain [35]. Motor neurons cultured from *Sacs*−/− embryos showed a comparable rearrangement of neurofilament bundles with elongated mitochondria and reduction in mitochondrial motility [35]. These observations suggest that disruption of mitochondrial organisation and dynamics, through alterations in cytoskeletal proteins, underpin the pathophysiological basis of ARSACS [35].

The reference sequence NM_014363.4 has ten exons, of which nine are coding. More than 180 mutations have now been reported in the *SACS* gene (LOVD, HGMD Pro), 80% of which are in exon 10. Approximately one quarter of all reported mutations are nonsense mutations, a third are missense, and the remainder comprise deletions and insertions of various sizes.

Homozygosity mapping is a valuable tool for the identification of defective loci, especially in patients born from consanguineous relationships [36], and has been successfully used in cases of ARCA, including ARSACS [37]. However, a high percentage of consanguineous ataxia families have been found to segregate with loci not corresponding to known ataxia genes [37,24], suggesting that further unidentified ARCA entities exist and paving the way for the discovery of novel causative genes [37].

By combining the homozygosity data obtained from the SNP array with the WES data, we were able to identify a single variant present in an identified region of LCSH (Figure 3). The 2.7 M array additionally enabled the exclusion of inherited copy number variations above the size thresholds stated above, which would not be detected by WES. The authors note that the possibility of hemizygosity has not fully been excluded as parental DNA is not available for analysis and heterozygous exonic or multi-exonic deletion of the *SACS* gene has not been excluded. SNP or haplotype analysis of the region could be of value in determining whether the identified mutation originated from English or Maori ancestry; however, unavailability of resources prevented this analysis from being undertaken.

WES has become an increasingly powerful and affordable tool in the diagnostic setting owing to recent developments in high-throughput sequence capture methods and next-generation sequencing approaches. Aside from identifying novel genes causative of rare disorders, it provides a time- and cost-efficient means of detecting mutations in genes already implicated in disease, especially diseases with great genetic heterogeneity [38]. Obtaining a specific molecular diagnosis facilitates tailored genetic counselling, enables carrier and prenatal testing to be offered and supports the development of therapeutic strategies.

In conclusion, we demonstrate that high-density SNP genotyping and WES provide an affordable and relatively quick approach for diagnostic laboratories to establish the molecular diagnosis in cases of genetically heterogeneous disorders. Our findings also expand the phenotypic variability and underlying genetic aetiology of ARSACS.

## References

[1] Anheim M., Tranchant C., Koenig M. (2012). The autosomal recessive cerebellar ataxias. N. Engl. J. Med..

[2] Hamza W., Pacha L.A., Hamadouche T., Muller J., Drouot N., Ferrat F., Makri S., Chaouch M., Tazir M., Koenig M. (2015). Molecular and clinical study of a cohort of 110 Algerian patients with autosomal recessive ataxia. BMC Med. Genet..

[3] Embiruçu E.K., Martyn M.L., Schlesinger D., Kok F. (2009). Autosomal recessive ataxias: 20 Types, and counting. Arq. Neuropsiquiatr..

[4] Gros-Louis F., Dupré N., Dion P., Fox M.A., Laurent S., Verreault S., Sanes J.R., Bouchard J.P., Rouleau G.A. (2007). Mutations in *SYNE1* lead to a newly discovered form of autosomal recessive cerebellar ataxia. Nat. Genet..

[5] Engert J.C., Bérubé P., Mercier J., Doré C., Lepage P., Ge B., Bouchard J.P., Mathieu J., Melançon S.B., Schalling M. (2000). ARSACS, a spastic ataxia common in northeastern Quebec, is caused by mutations in a new gene encoding an 11.5-kb ORF. Nat. Genet..

[6] Uziel T., Savitsky K., Platzer M., Ziv Y., Helbitz T., Nehls M., Boehm T., Rosenthal A., Shiloh Y., Rotman G. (1996). Genomic organization of the *ATM* gene. Genomics..

[7] Cytogenetics Assay Protocol. http://media.affymetrix.com/support/downloads/manuals/cyto_assay_usermanual.pdf.

[8] UCSC Genome Browser. http://genome.ucsc.edu.

[9] SNP Check 3. https://secure.ngrl.org.uk/SNPCheck/snpcheck.htm;jsessionid=5DDF10FED54806045E1D1F12B34F3017.

[10] Love J.M., Prosser D., Love D.R., Chintakindi K.P., Dalal A.B., Aggarwal S. (2014). A novel glycine decarboxylase gene mutation in an Indian family with nonketotic hyperglycinemia. J. Child. Neurol..

[11] ExAC Browser. http://exac.broadinstitute.org.

[12] HGMD Pro. https://portal.biobase-international.com/cgi-bin/portal/login.cgi?redirect_url=/hgmd/pro/start.php.

[13] Mutalyzer 2.0.13 Name Checker. https://mutalyzer.nl/name-checker.

[14] Bouchard J.P., Barbeau A., Bouchard R., Bouchard R.W. (1978). Autosomal recessive spastic ataxia of Charlevoix-Saguenay. Can. J. Neurol. Sci..

[15] Richter A., Rioux J.D., Bouchard J.P., Mercier J., Mathieu J., Ge B., Poirier J., Julien D., Gyapay G., Weissenbach J. (1999). Location score and haplotype analyses of the locus for autosomal recessive spastic ataxia of Charlevoix-Saguenay, in chromosome region 13q11. Am. J. Hum. Genet..

[16] El Euch-Fayache G., Lalani I., Amouri R., Turki I., Ouahchi K., Hung W.Y., Belal S., Siddique T., Hentati F. (2003). Phenotypic features and genetic findings in sacsin-related autosomal recessive ataxia in Tunisia. Arch. Neurol..

[17] Criscuolo C., Banfi S., Orio M., Gasparini P., Monticelli A., Scarano V., Santorelli F.M., Perretti A., Santoro L., De Michele G. (2004). A novel mutation in *SACS* gene in a family from southern Italy. Neurology.

[18] Vermeer S., Meijer R.P., Pijl B.J., Timmermans J., Cruysberg J.R., Bos M.M., Schelhaas H.J., van de Warrenburg B.P., Knoers N.V., Scheffer H. (2008). ARSACS in the Dutch population: A frequent cause of early-onset cerebellar ataxia. Neurogenetics.

[19] Ouyang Y., Sergers K., Bouquiaux O., Wang F.C., Janin N., Andris C., Shimazaki H., Sakoe K., Nakano I., Takiyama Y. (2008). Novel *SACS* mutation in a Belgian family with sacsin-related ataxia. J. Neurol. Sci..

[20] Crisculo C., Saccà F., De Michele G., Mancini P., Combarros O., Intante J., Garcia A., Banfi S., Filla A., Berciano J. (2005). Novel mutation of *SACS* gene in a Spanish family with autosomal recessive spastic ataxia. Mov. Disord..

[21] Richter A.M., Ozgul R.K., Poisson V.C., Topaloglu H. (2004). Private *SACS* mutations in autosomal recessive spastic ataxia of Charlevoix-Saguenay (ARSACS) families from Turkey. Neurogenetics..

[22] (2013). Novel *SACS* mutations identified by whole exome sequencing in a Norwegian family with autosomal recessive spastic ataxia of Charlevoix-Saguenay. PLoS ONE.

[23] Takado Y., Hara K., Shimohata T., Tokiguchi S., Onodera O., Nishizawa M. (2007). New mutation in the non-gigantic exon of *SACS* in Japanese siblings. Mov. Disord..

[24] Anheim M., Fleury M., Monga B., Laugel V., Chaigne D., Rodier G., Ginglinger E., Boulay C., Courtois S., Drouot N. (2010). Epidemiological, clinical, paraclinical and molecular study of a cohort of 102 patients affected with autosomal recessive progressive cerebellar ataxia from Alsace, Eastern France: Implications for clinical management. Neurogenetics.

[25] Shimazaki H., Takiyama Y., Sakoe K., Ando Y., Nakano I. (2005). A phenotype without spasticity in sacsin-related ataxia. Neurology.

[26] Shimazaki H., Sakoe K., Niijima K., Nakano I., Takiyama Y. (2007). An unusual case of a spasticity-lacking phenotype with a novel *SACS* mutation. J. Neurol. Sci..

[27] Baets J., Deconinck T., Smets K., Goossens D., Van den Bergh P., Dahan K., Schmedding E., Santens P., Rasic V.M., Van Damme P. (2010). Mutations in *SACS* cause atypical and late-onset forms of ARSACS. Neurology.

[28] Verhoeven W.M., Egger J.I., Ahmed A.I., Kremer B.P., Vermeer S., van de Warrenburg B.P. (2012). Cerebellar cognitive affective syndrome and autosomal recessive spastic of Charlevoix-Saguenay: A report of two male sibs. Psychopathology.

[29] Mignarri A., Tessa A., Carluccio M.A., Rufa A., Stort E., Bonelli G., Marcotulli C., Santorelli F.M., Leonardi L., Casali C. (2014). Cerebellum and neuropsychiatric disorders: Insights from ARSACS. Neurol. Sci..

[30] Bouchard J., Richter A., Melancon S., Mathieu J., Michaud J., Klockgether K. (2000). Autosomal recessive spastic ataxia (Charlevoic-Saguenay). Handbook of Ataxia Disorders.

[31] Martin M.H., Bouchard J.P., Sylvain M., St-Onge O., Truchon S. (2007). Autosomal recessive spastic ataxia of Charlevoic-Saguenay: A report of MR imaging in 5 patients. Am. J. Neuroradiol..

[32] Engert J.C., Doré C., Mercier J., Ge B., Bétard C., Rioux J., Owen C., Bérubé P., Devon K., Birren B. (1999). Autosomal recessive spastic ataxia of Charlevoix-Saguenay (ARSACS): High-resolution physical and transcript map of the candidate region in chromosome region 13q11. Genomics.

[33] Mrissa N., Belal S., Hamida C.B., Amouri R., Turki I., Mrissa R., Hamida M.B., Hentati F. (2000). Linkage to chromosome 13q11–12 of an autosomal recessive cerebellar ataxia in a Tunisian family. Neurology.

[34] Parfitt D.A., Michael G.J., Vermeulen E.G., Prodromou N.V., Webb T.R., Gallo J.M., Cheetham M.E., Nicoll W.S., Blatch G.L., Chapple J.P. (2009). The ataxia protein sacsin is a functional co-chaperone that protects against polyglutamine-expanded ataxin-1. Hum. Mol. Genet..

[35] Larivière R., Gaudet R., Gentil B.J., Girard M., Conte T.C., Minotti S., Leclerc-Desaulniers K., Gehring K., McKinney R.A., Shoubridge E.A. (2015). *Sacs* knockout mice present pathophysiological defects underlying autosomal recessive spastic ataxia of Charlevoic-Saguenay. Hum. Mol. Genet..

[36] Ben Hamida M., Belal S., Sirugo G., Ben Hamida C., Panayides K., Ionannou P., Beckman J., Mandel J.L., Hentati F., Koenig M. (1993). Friedreich’s ataxia phenotype not linked to chromosome 9 and associated with selective autosomal recessive vitamin E deficiency in two inbred Tunisian families. Neurology.

[37] H’mida-Ben Brahim D., M’zahem A., Assoum M., Bouhlal F., Fattori F., Anheim M., Ali-Pacha L., Ferrat F., Chaouch M., Lagier-Tourenne C. (2011). Molecular diagnosis of known recessive ataxias by homozygosity mapping with SNP arrays. J. Neurol..

[38] Singleton A.B. (2011). Exome sequencing: a transformative technology. Lancet Neurol..

